# Association of Serum C-Peptide Concentrations with Cancer Mortality Risk in Pre-Diabetes or Undiagnosed Diabetes

**DOI:** 10.1371/journal.pone.0055625

**Published:** 2013-02-06

**Authors:** Chih-Neng Hsu, Chia-Hsuin Chang, Yu-Sheng Lin, Jou-Wei Lin, James L. Caffrey

**Affiliations:** 1 Cardiovascular Center, National Taiwan University Hospital Yun-Lin Branch, Dou-Liou City, Yun-Lin County, Taiwan; 2 Department of Medicine, College of Medicine, National Taiwan University, Taipei, Taiwan; 3 Department of Internal Medicine, National Taiwan University Hospital, Taipei, Taiwan; 4 Department of Environmental and Occupational Health, University of North Texas Health Science Center, Fort Worth, Texas, United States of America; 5 Department of Integrative Physiology and Cardiovascular Research Institute, University of North Texas Health Science Center, Fort Worth, Texas, United States of America; University of Porto, Portugal

## Abstract

**Background:**

Known associations between diabetes and cancer could logically be attributed to hyperglycemia, hypersecretion of insulin, and/or insulin resistance. This study examined the relationship between initial glycemic biomarkers among men and women with impaired fasting glucose or undiagnosed diabetes and cancer mortality during follow up.

**Methods:**

The cohort included subjects aged 40 years and above from the Third National Health and Nutrition Examination Survey (NHANES III) with fasted serum glucose >100 mg/dl without the aid of pharmaceutical intervention (insulin or oral hypoglycemics). Cancer mortality was obtained from the NHANES III-linked follow-up database (up to December 31, 2006). A Cox regression model was applied to test for the associations between cancer mortality and fasting serum glucose, insulin, glycosylated hemoglobin (HbA1c), C-peptide, insulin like growth factor (IGF-1), IGF binding protein 3 (IGFBP3) and estimated insulin resistance.

**Results:**

A total of 158 and 100 cancer deaths were recorded respectively from 1,348 men and 1,161 women during the mean 134-month follow-up. After adjusting for the effect of age and smoking in women, all-cause cancer deaths (HR: 1.96 per pmol/ml, 95% CI: 1.02–3.77) and lung cancer deaths (HR: 2.65 per pmol/ml, 95% CI: 1.31–5.36) were specifically associated with serum C-peptide concentrations. Similar associations in men were not statistically significant. Serum glucose, HbA1c, IGF-1, IGFBP3 and HOMA were not independently related to long-term cancer mortality.

**Conclusion:**

C-peptide analyses suggest a modest association with both all-cause and lung cancer mortality in women but not in men. Further studies will be required to explore the mechanisms.

## Introduction

The association between type 2 diabetes and several types of cancer has been widely reported [1]. Diabetes and cancer share many common risk factors such as age, sex, race, socioeconomic status, body mass index, insulin resistance, physical activity, smoking, and alcohol intake [2]. These earlier analyses are complicated by a variety of therapeutic interventions (insulin, metformin, oral hypoglycemics, angiotensin receptor antagonists, statins, etc.) commonly employed in diabetic therapy that also may influence the incidence of cancer [3], [4], [5], [6].

The extended debate regarding mechanisms that link type 2 diabetes and cancer remains unresolved. The mitogenic effects of elevated insulin and the energetic effects of elevated glucose were logical candidates as risk factors for cancer [7], [8]. However, there is no similar risk of cancer associated with type 1 diabetes, suggesting that hyperglycemia per se is not the primary factor [1]. Furthermore, within type 2 diabetic patients, aggressive versus standard glycemic control does not appear to reduce cancer risk [9]. These results indicate that elevated insulin secretion is perhaps the better mechanistic candidate than hyperglycemia. In support of this thesis, evidence suggests that the mechanisms underlying the association between pre-diabetes/metabolic syndrome and cancer incidence involves the influence of elevated insulin and IGF-1 [10]. Likewise, associations have been proposed between IGF-1 and its binding protein IGFBP3 with specific tumor stages and grades at diagnosis and the resulting risk of recurrence and mortality [11]. However, specific associations between hyperglycemia, insulin, IGF-1, IGFBP3 and the risk of cancer among people with type 2 diabetes remain unclear.

Thus, this study was designed to test the hypothesis that one or more early glycemic biomarkers for type 2 diabetes are specifically associated with cancer mortality on follow-up among the middle-aged men and women with impaired fasting glucose (IFG) or undiagnosed diabetes in the general US population. To accomplish this aim, a collection of initial glycemic biomarkers (hyperglycemia, insulin secretion, insulin resistance, etc.) were analyzed for independent associations with long-term cancer outcomes within a nationally representative sample assembled from the Third National Health and Nutrition Examination Survey (NHANES III).

## Materials and Methods

### Participants

The Third National Health and Nutrition Examination Survey (NHANES III), conducted by the National Center for Health Statistics (NCHS) and the Centers for Disease Control and Prevention from 1988 through 1994, was the seventh in a series of surveys based on a complex, multi-stage sample design [12]. The NHANES III was approved by the NCHS Institutional Review Board. The current analysis was restricted to the adults aged 40 years and above with an impaired fasting blood glucose (IFG) or undiagnosed diabetes. IFG was defined as a fasted serum glucose >100 mg/dl without insulin or oral hypoglycemic therapy and undiagnosed diabetes defined as fasted serum glucose >126 mg/dl similarly without pharmacologic intervention. Race/ethnicity was categorized to non-Hispanic white, non-Hispanic black, and Mexican American. Race/ethnicity categorized as “others” was excluded from the analysis. Participants with previous history of malignancy or missing following-up information were also excluded.

### Anthropometric and Biochemical Data

Data were collected at all study sites by trained personnel according to standardized procedures. Social and demographic information such as age, sex, and race/ethnicity was collected during household interviews [13]. Laboratory measurements were performed in a mobile examination center [14]. Plasma glucose concentrations (mg/dl) were determined by the hexokinase method. Serum insulins (uU/mL) were determined by radioimmunoassay (RIA). Insulin resistance (IR) was estimated using the homeostasis model assessment: HOMA-IR = insulin (µU/mL) x glucose (mmol/L)/22.5 [15]. Glycosylated hemoglobin (HbA1c) measurements were performed by the Diabetes Diagnostic Laboratory at the University of Missouri - Columbia using the Diamat Analyzer System (Bio-Rad Laboratories, Hercules, CA). C-peptide (pmol/mol), a measure of endogenous insulin secretion, was also measured by RIA (Bio-Rad Laboratories) [16]. Body mass index (BMI) was defined as body mass (kg) divided by the height squared (m^2^). Subjects with serum cotinine values greater than 14 ng/ml were classified as current smokers, otherwise as nonsmokers [17].

### Attainment of Cancer Mortality

Of the adult NHANES III participants aged 40 and above, 99.9% were eligible for mortality follow-up by linkage with the National Death Index [18]. All-cause cancer (ICD codes: C00–C95) and lung cancer (ICD codes: C33–34) were analyzed for men and women, respectively. Follow-up for each participant was calculated as the difference between the NHANES III examination date and the end of follow-up (date of death or December 31, 2006, whichever occurred first). Those found alive were right-censored at the last date known alive or at the end of the follow-up. For lung cancer analysis, those who died from other cancers were also right-censored at the time of death [19]. The sample sizes for other specific cancers were considered insufficient to warrant individual analyses.

### Statistical Analysis

Continuous variables were reported as median and interquartile range. Categorical data were reported as percentages and standard error of the mean (SE). The cancer mortality analysis includes both cancer mortality and a subset of cancer incidence. A Cox proportional hazard model was used to calculate the hazard ratios (HR) in the evaluation of cancer mortality risk associated with fasted serum glucose, HbA1c, C-peptide, insulin resistance for all cancer and specifically for lung cancer mortality. Other site-specific cancers, such as colorectal cancer, prostate cancer in men, and breast cancer in women were not further evaluated due to limited mortality sample sizes. As widely accepted predictors of cancer outcomes, age and smoking status were added into the model as covariate adjustments, as well as race/ethnicity and body mass index [20], [21].

Due to the differences in cancer epidemiology among men and women, the cancer mortality analysis was stratified by sex. A sample weight was thus used to adjust for the unequal probabilities of selection to represent the U.S. population [14]. Hazard ratios and 95% confidence interval were reported. Statistical analyses were conducted using IBM SPSS Statistics 17.0 (SPSS Inc., Chicago, IL) accounting for complex survey design. A two-tailed p-value less than 0.05 was regarded as statistically significant.

### Auxiliary Analysis

The NHANES III survey updated the data on insulin like growth factor (IGF-1) and IGF binding protein-3 (IGFBP3) in October 2006. Both of these proteins have been the subject of attention due to potential associations with cancers [22]. IGF-1 (ng/ml) and IGFBP3 (ng/ml) were tested in 6,061 serum samples from adults aged 20 or older who attended the morning session of the examination after an overnight fast during NHANES III. The determinations were made using standard laboratory protocols described by Diagnostic Systems Laboratories Inc (DSL, Webster TX) [23]. Thus, only a proportion of the reports for adults included in the main analysis included data for IGF-1 and IGFBP3. The two variables were dichotomized. The missing values of IGF-1 and IGFBP3 in some of the included subjects were analyzed as the third category “missing” in addition to “upper half” and “lower half”. The two new categorical variables were added into the Cox regression model, in addition to fasted serum glucose, HbA1c, C-peptide, insulin resistance, age, smoking, and race/ethnicity. The association between the categories of IGF-1, IBGBP3 and all-cause cancer mortality and lung cancer mortality were determined.

## Results

A total of 1,348 men and 1,161 women aged 40 years and above with pre-diabetes or undiagnosed diabetes were included for analysis. The baseline demographic characteristics of participants stratified by sex are summarized in Table 1. During an average of 134 months of follow-up (median: 155 months, range 0–218 months), there were 158 cancer deaths for men and 100 for women. Among them, lung cancer deaths occurred in 42 men and 16 women. Other specific cancers were not analyzed due to limited sample numbers.

**Table 1:** Demographic characteristics of subjects – NHANES III, 1988–1994.

Parameters^a^	Men (n = 1,348)	Women (n = 1,161)
	Median (Interquartile range)	Median (Interquartile range)
Age (yrs)	57.9 (47.2–68.6)	60.9 (49.8–72.0)
Body mass index (kg/m^2^)	27.5 (25.1–30.8)	27.9 (23.8–32.0)
Fasted serum glucose (mg/dl)	107 (103–117)	107 (103–116)
Glycated hemoglobin (%)	5.51 (5.16–5.98)	5.56 (5.25–5.94)
HOMA insulin resistance index^b^	3.14 (2.18–4.72)	3.24 (2.25–5.04)
Serum C-peptide (pmol/ml)	0.93 (0.61–1.31)	0.95 (0.64–1.30)
Insulin Like Growth Factor-I (ng/ml)^c^	232 (180–281)	190 (153–233)
IGFBP3 (ng/ml)^c^	4147 (3933–4801)	4397 (3755–4983)
Race/ethnicity	Percentage (SE)	Percentage (SE)
Mexican American	4.97 (0.61)	4.38 (0.46)
Non-Hispanic black	9.14 (1.02)	11.6 (1.26)
Non-Hispanic white	85.9 (1.33)	84.1 (1.42)
Smoking status (current smoker)^d^	30.3 (1.81)	21.5 (2.01)

a All of the analyses were adjusted with the NHANES III sample weights. Analayis included paticipants aged 40 years and above who had fasted glucose greater than 100 mg/dl without any oral hypoglycemic agent or insulin.

b Calculated as insulin (µU/mL) x glucose (mmol/L)/22.5.

c Approximately 50% of IGF1 and IGFBP3 measurements are missing. Binary (high vs. low) values were determined separately by gender from weighted distribution of available data for both gender (n = 671 and 615 for men and women, respectively).

d Defined as serum cotinine level >14 ng/mL.

Abbreviations: IGF1, insulin like growth factor; IGFBP3, insulin like growth factor binding protein-3; HOMA, homeostasis model assessment - insulin resistance; SE, standard error;

In men, age and smoking were independently associated with all-cause cancer deaths and with lung cancer deaths. However, none of the following biomarkers, fasting serum glucose, HbA1c, HOMA-IR, and serum C-peptide, were related to either all-cause cancer deaths or to lung cancer deaths (Table 2, upper panel).

**Table 2:** Cancer mortality analysis of risk factors associated with all-cause and lung cancer.

Men		
	All Cancers (n = 158)	Lung Cancer (n = 42)
	Hazard Ratio (95% CI)	Hazard Ratio (95% CI)
Age (yrs)	1.11 (1.09–1.13)*	1.08 (1.05–1.11)*
Body mass index (kg/m^2^)	1.01 (0.93–1.10)	0.91 (0.80–1.03)
Race/ethnicity		
Mexican American	0.75 (0.44–1.30)	0.44 (0.12–1.54)
Non-Hispanic black	0.91 (0.62–1.34)	0.77 (0.35–1.71)
Non-Hispanic white	1.00 (reference)	1.00 (reference)
Smoking (reference: nonsmokers)^a^	1.99 (1.16–3.41)*	3.91 (1.33–11.5)*
Fasted serum glucose (mg/dl)	1.00 (0.99–1.01)	0.99 (0.97–1.01)
Glycated hemoglobin (%)	1.24 (0.90–1.70)	1.53 (0.78–3.00)
HOMA insulin resistance index^b^	0.92 (0.80–1.05)	0.90 (0.68–1.18)
Serum C-peptide (pmol/ml)	1.25 (0.71–2.21)	1.62 (0.77–3.42)

Women		
	All Cancers (n = 100)	Lung Cancer (n = 16)
	Hazard Ratio (95% CI)	Hazard Ratio (95% CI)
Age (yrs)	1.06 (1.04–1.07)*	1.06 (1.03–1.09)*
Body mass index (kg/m^2^)	0.97 (0.93–1.02)	0.99 (0.89–1.09)
Race/ethnicity		
Mexican American	1.25 (0.71–2.22)	1.46 (0.16–13.1)
Non-Hispanic black	1.24 (0.76–2.02)	1.39 (0.39–4.97)
Non-Hispanic white	1.00 (reference)	1.00 (reference)
Smoking (reference: nonsmokers)^a^	1.71 (0.81–3.62)	7.08 (1.19–41.9)*
Fasted serum glucose (mg/dl)	1.00 (0.99–1.02)	1.02 (1.00–1.05)#
Glycated hemoglobin (%)	0.97 (0.57–1.65)	0.39 (0.14–1.04)
HOMA insulin resistance index^b^	0.96 (0.87–1.06)	0.99 (0.90–1.09)
Serum C-peptide (pmol/ml)	1.96 (1.02–3.77)*	2.65 (1.31–5.36)*

* p<0.05.

# p = 0.06.

a Defined as serum cotinine level >14 ng/mL.

b Calculated as insulin (µU/mL) x glucose (mmol/L)/22.5.

In women, age was a risk for both all-cause and lung cancer deaths (HR: 1.06, 95% CI: 1.04–1.07) however, the association between smoking and cancer mortality remained statistically significant for lung cancer (HR: 7.08, 95% CI: 1.19–41.9), but not for the larger category of all-cause cancers. In addition, for women only, there was a positive association between serum C-peptide concentrations and both all-cause cancer deaths (HR: 1.96 per pmol/ml, 95% CI: 1.02–3.77) and lung cancer deaths (HR: 2.65 per pmol/ml, 95% CI: 1.31–5.36). And, as with men, there was no statistically significant association between serum glucose, HbA1c, and HOMA-IR with either all-cause or lung cancer mortality. Of note, the relationship between fasting serum glucose and the risk of dying from lung cancer did approach statistical significance in women and may thus require more extensive future analysis in a larger cohort (HR: 1.02 per mg/dl, 95% CI: 1.00–1.05, p = 0.06) (Table 2, low panel).

In order to examine the likelihood of reverse causation, a time lag analysis was performed to exclude the occurrence of early deaths during the first five years. Using the same Cox regression model, the hazard ratio between serum C-peptide concentrations and all-cause cancer death was similar to that found in the original analysis (HR: 1.74, 95% CI: 0.85–3.56, p = 0.13), despite of borderline statistical significance. The association between serum C-peptide concentrations and lung cancer death remained significant in women (HR: 3.01, 95% CI: 1.26–7.17, p = 0.01).

Valid values for IGF-1 and IGFBP3 were available from only a sub-sample of 1,355 people. Neither IGF-1 nor IGFBP3 was associated with either all cancer death or lung death among men or women in the multivariate analysis (Table 3).

**Table 3:** Cancer mortality analysis of risk factors associated with all-cause and lung cancer mortality with integration of insulin like growth factor 1 and IGF binding protein 3 (IGFBP3).

Men^a^		
	All Cancers	Lung Cancer
	Hazard Ratio (95% CI)	Hazard Ratio (95% CI
Insulin Like Growth Factor 1 (ng/ml)		
High (>50^th^ percentile)	0.51 (0.25–1.03)	0.84 (0.19–3.68)
Low (<50^th^ percentile)	1	1
IGFBP3 (ng/ml)		
High (>50^th^ percentile)	1.32 (0.64–2.72)	1.32 (0.24–7.18)
Low (<50^th^ percentile)	1	1

Women^a^		
	All Cancers	Lung Cancer
	Hazard Ratio (95% CI)	Hazard Ratio (95% CI
Insulin Like Growth Factor 1 (ng/ml)		
High (>50^th^ percentile)	1.40 (0.51–3.85)	0.57 (0.02–14.73)
Low (<50^th^ percentile)	1	1
IGFBP3 (ng/ml)		
High (>50^th^ percentile)	0.71 (0.29–1.76)	2.00 (0.09–44.87)
Low (<50^th^ percentile)	1	1

a All of the analyses are adjusted with sampling weight. Covariates include age, body mass index, race/ethnicity, smoking, fasted serum glucose, glycated hemoglobin, HOMA insulin resistance index, and serum C-peptide.

## Discussion

The results of this study have demonstrated that among glycemically vulnerable subjects, serum C-peptide concentration was associated with a modestly increased risk for long-term overall cancer mortality and lung cancer mortality in women. Since the majority of insulin is removed in the first pass through the hepatic circulation, C-peptide concentrations are presumed to better reflect insulin secretory rates than circulating insulin. A similar association between C-peptide and cancer mortality risk was not observed in men. Neither serum glucose nor estimated insulin resistance was related to long-term cancer outcomes in this selected cohort of subjects with pre-diabetes and undiagnosed diabetes. Though the increased risk associated with C-peptide is modest, the clinical outcome is serious and hyperinsulinemia includes a large and rapidly growing segment of our population.

A recent analysis on the NHANES 1988–1994 survey in which 15594 people (aged 20–89) with metabolic syndrome were followed an average of 8.5-years, demonstrated that for every 50 mg/dl increase in plasma glucose, there was a 22% increased risk of overall cancer mortality, and insulin resistance was associated with a 41% increased risk of overall cancer mortality [24]. However, the hazard ratios for C-peptide were not statistically significant in univariate analysis (HR: 1.05, 95% CI: 0.87–1.28) [24]. On the contrary, the current analysis incorporated an analytic method more appropriate to represent general U.S. population of untreated pre-diabetics and undiagnosed diabetics. In the prior study, the inclusion of younger presumably healthier subjects (aged 20–40 years) and shorter follow up (mean: 8.5 years) may have diluted and/or obscured some outcomes. This current cohort of middle aged and older subjects (aged≥40 years) are typically more likely to develop cancer and the somewhat longer follow-up period (median: 13 years) provides more opportunity to determine its influence on mortality. The current analysis suggests that when C-peptide, HOMA-IR, serum glucose and HbA1c are considered simultaneously, markers of insulin secretion (C-peptide) appear to be the major determinant for future cancer mortality but only in women.

The results of the analysis above imply that C-peptide concentrations may help to identify a risk of long-term cancer mortality in women prior to the appearance of overt symptoms of malignancy. Prior analyses from our group suggest that the metabolic syndrome poses a significant increase in non-cardiovascular mortality risk primarily in postmenopausal women and not in men [21]. These observations begin to suggest a sex-specific cancer risk associated with metabolic dysregulation. Similarly, an interaction between visceral fat, sex hormone, and inflammatory responses might be related to the differential association between C-peptide and cancer outcomes between men and women [25], [26]. The Health, Eating, Activity, and Lifestyle (HEAL) Study demonstrated that women with high fasted C-peptide concentrations (values>2.5 ng/mL) collected 3 years after cancer diagnosis had more than a two-fold increased risk of breast cancer death compared to those with low C-peptide measurements [27]. The relationship between post-diagnosis C-peptide and breast cancer death rates was also found in women with early breast cancer [28]. The current analysis suggests that the association of C-peptide with future cancer mortality might be extended to very early in the progression when serum glucose levels first begin to exceed 100 mg/dl in women.

C-peptide is a direct biomarker of endogenous insulin secretion. The link between C-peptide, hyperinsulinemia, other metabolic disturbances and cancer risk is very likely complex and the precise mechanisms involved remain largely unknown [29]. Despite reports of various effects of obesity on IGFBP3, C-peptide and IGF-1, BMI was not independently associated with cancer outcomes once the effect of serum C-peptide was adjusted [30]. That finding might suggest that the influence of obesity on cancer mortality is mediated by excess insulin secretion. Whether insulin secretion modifies cancer development or simply the mortality risk (prognosis) is not clear from the current analyses. Thus, treatment strategies, including weight-loss, physical activity, and insulin-lowering medications are logical strategies to reduce insulin secretion, C-peptide and future cancer risk [27].

The pathway linking insulin production to cancer outcomes independent of glycemic control and insulin resistance is suggested to involve tumorigenesis driven by increased insulin, IGF 1 and IGF 2, collectively signaling through both insulin and IGF-1 receptors [31]. Recent epidemiologic studies suggest that IGF-1 is associated with obesity and cancer [32]. IGF-1 concentrations were reported as influenced by age, adiposity, serum glucose, and metabolic syndrome [33]. Despite this collective evidence, the auxiliary analysis above did not suggest that IGF-1 is an independent risk factor or an intervening factor for long-term cancer mortality.

Despite the general agreement that obesity is a strong predictor for insulin resistance [34], the current analyses did not identify any independent relationship between BMI and cancer mortality once C-peptide concentration was incorporated into the model. One feasible explanation is that it is insulin secretion and not insulin resistance which is key. This is supported perhaps by the absence of an association between cancer mortality and HOMA in the current analyses. Insulin is an important if not the most important growth factor. Insulin resistance may provide a protective shield for most tissues and perhaps some cancers or some stages in their progression escape the evolving insulin resistance associated with obesity or type 2 diabetes. Finally high serum C-peptide may have an unappreciated influence of its own independent of insulin.

The exclusion of cancer deaths identified in the early follow-up helped to eliminate the potential influence of undiagnosed cancer cases at baseline. The association between serum C-peptide concentrations and all-cause/lung cancer death in women remained similar to those estimated from the original analysis. The results from the time-lag analysis helps to confirm the temporal sequence in the associations of interest and make the possibility of reverse causation less likely [35].

The first limitation of the current analysis is that IGF-1 and IGFBP3 were available on only a small portion of the NHANES data set. Thus, the power to demonstrate the effect of IGF-1 and IGFBP3 may have been insufficient as mentioned above. Moreover, the analysis relied on single one time measurements for each of the glycemic markers. Those adults with untreated diabetes and serum glucose values greater than 100 mg/ml were very likely to have subsequently been treated with insulin, oral hypoglycemics or other drugs. Such treatment may have moderated the risk during the follow-up period. The effect of subsequent oral hypoglycemic agents and insulin could not be accounted for in the current analysis. Furthermore, the analysis was restricted to men and women aged 40 years and above characterized as pre-diabetics and unrecognized diabetics but not currently exposed to any anti-diabetic medication. Despite selecting a presumably cancer-prone sub-population, the number of cancer deaths was still too small to develop a reliable prediction model. The final analysis set did not have sufficient power to evaluate the association with specific cancers other than those of the lung. Finally, the association between C-peptide and cancer mortality is modest at best. The hazard ratio is 1.96 per each increment of 1 pmol/mL in C-peptide. But the interquartile range in the population is only 0.7 pmol/ml (0.61–1.31), so the relative risk from 25th to 75th percentile is low. The association was not found in men, thus, differences in sex hormones may play a role. This is of course speculation and the mechanism is unclear.

In conclusion, the results of this study have demonstrated that circulating C-peptide concentrations are associated in women with a modest risk of both long-term all-cause cancer mortality and in this limited cohort specifically with mortality from lung cancer. Similar associations were not found in men. The relationship of C-peptide with cancer at other specific sites could not be reliably examined due to limited analyte sample numbers and site specific mortality events. Additional basic and clinical studies will be required to further validate the current findings and explore the mechanisms underlying the associations of interest.
